# Snare-assisted double pancreatic duct stenting for severe pancreatic stricture

**DOI:** 10.1055/a-2924-6696

**Published:** 2026-08-11

**Authors:** Kazuya Sumi, Jun Ushio, Tomoya Sato, Hisaki Kato, Yuki Kawasaki, Takayoshi Ito, Haruhiro Inoue

**Affiliations:** 1 Digestive Diseases Center 378609 Showa Medical University Koto Toyosu HospitalKoto-kuTokyoJapan

Pancreatic duct (PD) strictures associated with chronic pancreatitis (CP) are often difficult to manage endoscopically. In severe fibrotic strictures, sufficient working space for pancreatoscopy-guided lithotripsy of pancreatic stones may not be obtained, and stepwise PD dilation using multiple stents may be required. 1​ ​2​ Additional stent placement can be technically challenging because device advancement through tight strictures may push the initially placed stent distally.

A man in his 50s with CP presented with abdominal pain associated with pancreatolithiasis and severe main PD stricture. Endoscopic treatment was planned. Because transpapillary access through the major papilla was difficult, initial PD drainage had been achieved via the minor papilla using a 7-Fr plastic stent.

Balloon dilation enabled the insertion of a pancreatoscope, but the severe stricture prevented adequate maneuverability and sufficient working space for lithotripsy. Therefore, staged PD dilation using multiple plastic stents was planned before reattempting lithotripsy.

Double guidewire placement across the stricture was achieved, and sequential insertion of 7-Fr and 5-Fr PD stents was attempted. Advancement of the additional device pushed the initially placed stent distally despite grasping with biopsy forceps, and additional stenting could not be completed.

**Fig. 1:**
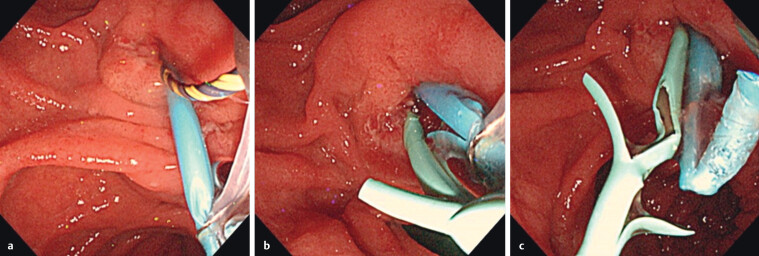
( **a** ) A guidewire was advanced while stabilizing the initially placed pancreatic duct (PD) stent with a thin snare. ( **b** ) An additional PD stent was advanced while maintaining snare fixation of the initial stent. ( **c** ) Successful placement of the additional PD stent without distal migration.

Therefore, a thin snare was used to grasp and stabilize the initially placed stent. While maintaining traction and axis stability, the additional PD stent was successfully advanced alongside the initial stent without distal migration ( Fig. 1 and Video 1 ).

**Video 1** Snare-assisted double pancreatic duct stenting for severe pancreatic duct stricture, enabling the safe placement of an additional stent without distal migration.


Snare-assisted techniques for migrated pancreatic and biliary stents have recently been reported. 3​ 4​ ​5​ In the present case, the same concept was applied to stabilize the initially placed stent and prevent distal migration during additional PD stenting. Snare-assisted fixation provided stable traction and axis control, allowing the safe advancement of an additional PD stent without specialized devices. This technique may represent a simple and reproducible option for severe fibrotic PD strictures.

Endoscopy_UCTN_Code_TTT_1AR_2AZ
